# Effects of word length, contextual support, and prior second language proficiency on the learning of technical words in a second language

**DOI:** 10.1371/journal.pone.0334038

**Published:** 2025-10-06

**Authors:** Jie Wang, Yen Na Yum

**Affiliations:** 1 Department of Psychology, The Education University of Hong Kong, Hong Kong SAR, China; 2 Department of Special Education and Counselling, The Education University of Hong Kong, Hong Kong SAR, China; University of Padova, ITALY

## Abstract

Second language (L2) learners often struggle with learning technical words in a new discipline. Previous studies suggested that word length and context may differentially affect orthographic and semantic acquisition during initial L2 word learning. This study investigated how (1) target word length, (2) contextual support, and (3) learners’ L2 vocabulary and spelling abilities influence orthographic and semantic recognition in initial acquisition of L2 technical words. Eighty-eight Chinese-English university students read sentences with high- and low-constraint contexts about sixteen L2 English technical words and then performed orthographic and semantic recognition tasks. Recognition accuracy was examined through multiple-choice questions with fully and partially correct answer choices and analyzed with generalized linear mixed-effects models. Results showed that longer word length impaired orthographic recognition but facilitated partial semantic recognition, while contextual support enhanced semantic but not orthographic learning of L2 technical words. Skilled spellers performed better on orthographic recognition than poorer spellers, while those with larger L2 vocabulary size also showed better orthographic learning, especially for shorter word forms. Hence, word length, contextual support, and learners’ L2 vocabulary and spelling abilities differentially affected orthographic and semantic learning of L2 technical words, and measurement of partial recognition facilitated a nuanced understanding of learning outcomes. The findings highlight the importance of adopting targeted pedagogical approaches to enhance L2 technical word learning.

## Effects of word length, contextual support, and prior second language proficiency on the learning of technical words in a second language

Knowledge of academic vocabulary is essential to understanding and using academic discourse. General academic vocabulary is commonly used in academic writing across various disciplines and has a broad application. For example, “analyze” and “paradigm” can be applied in various subject areas. Technical words, also called specialized vocabulary, are distinct in that they are “very frequent within a particular topic or discipline but less frequent outside that area” [1]. Examples of technical words in the medical disciplines include “biopsy” and “incision.” Technical words enable precise communication of domain-specific knowledge in specialized fields and make up a high proportion of discipline-specific texts. For instance, 37.6% of words in an anatomy text and 16.3% of words in an applied linguistics text were classified as technical words [2]. Learners who recognize and understand concepts represented by technical words can build connections in disciplinary knowledge [3]. Indeed, technical word knowledge was associated with specialized knowledge and overall academic performance [4]. Globally, many higher education institutions use English as a medium of instruction, and so non-native speakers of English need to use academic language in English as a second language (L2). However, English as a second language (ESL) university students in Hong Kong cited learning technical words as a core academic difficulty [5]. A growing number of Chinese ESL students in mainland China faces a similar situation. Previous experimental research also showed that learning technical words in L2 was more demanding and less effective than learning the same concepts in a first language (L1) [6–7].

While some research has examined how learners acquire general L2 words through reading authentic texts [8], few studies have delved into the learning of L2 technical words, which do not typically have correspondence in the L1 lexicon and are difficult to contextualize. Different factors at the word, context, and learner levels may impact how easily L2 technical words are learned. Investigating how these factors influence different aspects of L2 technical word learning would deepen our understanding of the interplay between linguistic and cognitive processes involved in vocabulary acquisition.

The present study adopted an experimental approach to investigate the initial learning of L2 technical words’ orthography and semantics among Chinese-English university students. We examined the effects of word length, semantic constraint of the sentence contexts, and L2 vocabulary knowledge and spelling skill on orthographic and semantic recognition, bringing together different levels of influence. Orthographic and semantic recognition here referred to the foundational reading processes that readers engage in to recognize the visual form and extract semantic information from visually presented words [9]. To understand how test items can reflect the accumulation of word knowledge, the learners’ test responses were categorized into full or partial recognition for both orthographic and semantic recognition. This knowledge could contribute to theoretical models of vocabulary learning and bilingual lexicon in specialized contexts. Practically, unraveling the initial processes of learning technical words could provide insights for language educators and curriculum developers to formulate learning and assessment strategies and cater to differences associated with L2 learning. This has particular significance for the teaching and learning of English for academic and specific purposes and serves as a basis for developing students’ ability to meaningfully participate in academic communities in multilingual contexts.

## Literature review

The ease and outcomes of L2 word learning are influenced by many target word characteristics, such as word frequency [10], orthographic and semantic similarity with L1 [11], and word length [12–13]. In general, L2 technical words are unfamiliar to learners since these words are rarely taught in typical language classrooms or seen in casual reading. They are also distinguished from general low-frequency words in that they appear in specific academic contexts and may require background knowledge of the discipline to understand. Explicit definitions and contextual usage are effective in clarifying and distinguishing between related concepts [14]. Several studies have also examined learner characteristics, such as prior knowledge of L2 [15]. For learners whose L1 belongs to another language family, such as Chinese, the distinct orthographic system of the L1 may also impact the orthographic processing of L2 words [16]. The following sections outline how word length and contextual support may differentially affect orthographic and semantic learning, and then highlight the effects of prior L2 knowledge on the learning process.

### Word length effects *on* L2 word learning

In experimental memory research, the classic word length effect refers to the better recall of lists of short words compared to lists of long words [17–18]. The word length of a target L2 word can be measured in print by counting the number of graphemes (e.g., letters) or in auditory form as the number of phonemes or syllables. Several studies on L2 word learning have examined the relation between word length and learning performance, but have yielded inconsistent findings [12,13,19,20]. Ellis and Beaton required English-speaking university students to learn L2 German vocabulary and then complete German-to-English and English-to-German translation tests [13]. The length of L2 German target words (4−9 letters) was negatively correlated with the production performance in L1-to-L2 English-to-German translation (−0.52 ≥ *r*s ≥ −0.70). The detrimental effect of word length was less apparent in L2-to-L1 German-to-English translation which did not require L2 word production (−0.11 ≥ *r*s ≥ −0.34). Barcroft and Rott compared native English speakers’ L1-L2 paired association learning of two-syllable and three-syllable words in L2 German or L2 Spanish [12]. In the L1-to-L2 translation test after learning, the learners spelled more complete forms of two-syllable words than those of three-syllable words across the two L2s. Interestingly, when the researchers assigned scores to partly correct responses (e.g., 0.5 if producing half of the word), they found that the learners produced more partial forms of three-syllable words than those of two-syllable words. The results suggest that longer words are more difficult for learners to fully master and may result in partial learning. For technical word learning, Gablasova found a similar effect: longer word length (varying from 2 to 13 letters) was moderately negatively correlated to semantic recall for L1 and L2 English technical words learned via reading [7].

On the other hand, there have been reports of null effect of word length on L2 vocabulary learning [15] and positive effects of word length on learning performance [19–20]. For instance, Jin and Webb found that Chinese university students remembered long English vocabulary better than short ones after listening to recorded teacher speech [19]. The students completed a semantic recall test by writing down the L1 meanings of the L2 target words. The length of L2 target words was positively correlated with semantic recall performance in both the immediate and the delayed posttests. The researchers explained this pattern by the higher salience of longer spoken words. Puimège and Peters also reported a learning advantage in orthographic recall for long single words in their study of incidental learning of L2 words by audiovisual input (i.e., watching television), which may be due to the greater opportunities for learners to identify and internalize spelling and sound patterns in longer words [20].

In sum, word length appeared to have complex relationships with orthographic and semantic acquisition in studies of L2 word learning. The modality of language input may be a confounding factor that has led to different effects since previous studies generally reported poorer learning performance for longer words when using visual input but better performance for longer words when using auditory or audiovisual input. Word length might also differentially affect orthographic and semantic acquisition, e.g., impeding orthographic learning but facilitating semantic learning. Most studies used word knowledge tests that tapped into the form-meaning link (e.g., translation) and thus required both orthographic and semantic knowledge. To better differentiate between orthographic and semantic learning, the current study adopted two types of multiple-choice questions in the test phase, one tapping on orthographic knowledge only and the other on the form-meaning link. Moreover, distractors in the multiple-choice questions were designed to be of two difficulty levels (including partially correct answer choices) to reflect the extent of partial learning of word knowledge.

### Effects *of* contextual support *on* L2 word learning

Another crucial factor in L2 technical word learning is the establishment of semantic-rich representations. The learning process for L2 technical words may be different from general L2 word learning. For adult ESL learners, most non-technical L2 words, e.g., *pear*, can be linked to well-established concepts through L1 translation equivalents, e.g., *梨*, or other semantic representations, e.g., a drawing of a pear, as posited by the Revised Hierarchical Model [21]. In contrast, since L2 technical words usually refer to novel concepts that learners have not acquired before entering that discipline, learners cannot easily connect the new concept to existing concepts or a corresponding L1 lexical item. Instead, when encountering an unfamiliar technical word in L2, e.g., *achondrite*, learners need to create coherent new concepts from a definition or sentence context, e.g., *a type of stony meteorite that does not contain round grains*, and link the meaning of the new concept with the L2 word form. This process has been described as conceptually-mediated word learning [22].

Semantic representations of new words may be built up through processing explicit and detailed definitions [14]. Many studies have also shown that semantically constraining contexts can spur incidental learning of new L2 words [15,23–26]. Despite some differences in the manipulations of the contextual support (e.g., latent semantic analysis scores between prime and target words, likelihood of correct guessing based on sentence context) and varying L2 proficiency among the participants, extant results are consistent in that semantic recognition and recall are facilitated by contextual support. Sentences with high semantic constraints may lead to word knowledge gains even after a single exposure [23,26]. However, the extent of learning depends on the specific measures, and repetitions up to ten times could still result in learning gains [27].

Like general L2 word learning, multiple exposures to technical words in relevant contexts can facilitate learners to refine word knowledge and achieve long-term retention [10]. In the study of Kuipers et al. [22], university students learned sixteen L1 technical words by reading ten high-constraint definition sentences or low-constraint vague sentences for each word. For sentences with high semantic constraints, participants self-reported an increase in semantic word knowledge (“*I know the meaning of the word*”) while showing an increase in late frontal negativity of the event-related potentials (ERPs) to the target words during learning. However, no such behavioral or neural changes were found in the low-constraint condition. In the subsequent meaning recognition test, participants responded faster and more accurately to technical words learned under high semantic constraints. Therefore, the amplitude change of the late frontal negativity might index conceptually-mediated learning over multiple exposures to technical words in semantically constraining contexts.

Regarding orthographic acquisition, some studies showed that learning tasks with a semantics focus (e.g., sentence writing, synonym generation) decreased learners’ retention of the L2 word forms during initial learning [28–29]. This may be due to the distribution of cognitive resources between learning orthography and semantics during initial word learning. Few studies have examined how the semantic constraint of sentence context affects orthographic learning. Yi and colleagues presented participants with novel L2 words in different sentence contexts (informative vs. neutral) and found that semantic context had no direct influence on either orthographic or semantic recognition of the L2 target words [26]. However, the study only presented the target words once to examine eye movements at the very initial stage of incidental word learning. There is limited empirical evidence on how semantic contexts may affect the learning dynamics of L2 technical words’ orthography and semantics.

### Effects *of* L2 proficiency *on* L2 word learning

Previous research has often included L2 proficiency as a moderating factor in studies of L2 lexical processing or learning. According to the lexical quality hypothesis [30], detailed and specific representations of lexical constituents (i.e., orthography, phonology, and semantics) support word recognition and reading comprehension. The lexical quality of newly learned L2 words may partially depend on the L2 proficiency of the learner. In contextual learning, information about the target word is embedded in the context which contains additional words and ideas. Learners with higher general L2 proficiency may efficiently use semantic support given their higher ability to extract meaning from the context [8,24]. However, some studies have reported that L2 proficiency or prior L2 knowledge had no effect on novel L2 word learning [25,31]. Previous studies mainly adopted an overall measure of L2 proficiency, which tested composite skills, including vocabulary, syntactic structure, reading comprehension, and others. It is possible that the mixed reports could be disentangled with greater separation of L2 proficiency between groups or more sensitive L2 proficiency measures.

It is also likely that specific subskills are differentially related to orthographic and semantic learning of novel words. It is important to identify which prior L2 word knowledge and word-related skills may contribute to further learning of specific aspects of L2 vocabulary. Teachers may then pinpoint the appropriate L2 language skills to develop to facilitate technical word learning. However, few studies have explicitly tested the roles of different L2 subskills in novel word learning. In this study, we included two measures of prior L2 word knowledge, namely, vocabulary breadth and spelling skill, which were hypothesized to be related to semantic and orthographic learning of L2 technical words, respectively.

### The current study

The Ontogenesis Model of the L2 Lexical Representation regards fuzziness as a pervasive property of L2 lexical representations, especially for the initial stages of acquisition [32]. Research has also shown that accruement of word knowledge is largely incremental [12,33,34]. To advance the understanding of the learning process during initial acquisition of L2 technical words, the current study examined university students’ full and partial learning of orthographic and semantic word knowledge after repeated exposures to L2 technical words embedded in sentence contexts. Learning confidence was collected as a manipulation check to verify that participants perceived a difference in the two contextual support conditions. Recognition tests were created to differentiate between orthographic and semantic learning, as well as to reflect full and partial learning of word knowledge, by including distractors of two difficulty levels in each type of multiple-choice questions. Generalized linear mixed-effects models were adopted to address the following research question: How do (1) target word length, (2) contextual support, and (3) learners’ general L2 vocabulary knowledge and spelling skill influence the full and partial learning of orthographic and semantic word knowledge in initial acquisition of L2 technical words?

Based on previous findings, we expected the word length of L2 technical words to be negatively related to orthographic recognition [7], and longer words might show more partial orthographic learning than shorter words [12]. Longer words might increase semantic recognition performance, since they might be visually more distinct and thus might facilitate the establishment of the form-meaning link [19]. High semantic constraints might increase semantic recognition of L2 technical words [22] but might not influence orthographic recognition [26]. Further, we expected that learners’ L2 vocabulary knowledge might facilitate semantic learning, while L2 spelling skills might facilitate orthographic learning. The interactions between the three factors mentioned, word length, contextual support, and prior L2 skills, were also examined. The learning and testing materials, cleaned data, and analysis codes of the current study are available from the Open Science Framework repository: https://osf.io/fxqvr/?view_only=f4ae94c1230148c684154063f30dd913

## Materials and methods

### Participants

The present study included 88 sequential bilingual Chinese-English university students attending English-medium universities in Hong Kong and mainland China (25 males; age = 20.8 ± 2.0 years). Participants were either native Cantonese Chinese (n = 47) or Mandarin Chinese (n = 41) speakers. Participants studied various majors but did not major in the academic disciplines of the target words (i.e., astronomy, neurology, pharmacology, and physics). The research protocol was approved by the Human Research Ethics Committee of the corresponding author’s institution. Participants were recruited via advertisements on university websites and personal contacts. They gave written informed consent online and received monetary compensation for their time.

Participants self-reported the following language background information: (a) L2 English proficiency in listening, speaking, reading, and writing on a 5-point scale (e.g., 1 = “I can only speak simple words or phrases, such as *hello* and *thank you*”; 5 = “I can speak this language as well as an educated native speaker”), (b) ages when beginning to learn oral or written English, and (c) percentages of English use in four domains of daily life (e.g., 0–100% of time for communicating with family members, communicating with friends, for school/work, and for personal entertainment). Table 1 summarizes their English language background.

**Table 1:** Participants’ English Language Background.

	*N*	Mean	Standard Error
Self-rated English proficiency (max score: 5)	88	3.21	0.07
Age of acquisition (years)			
English speaking	87	6.06	0.38
English reading	87	6.13	0.32
Percentage of time using English	88	10.72%	0.98%
Vocabulary performance (LexTALE)	88	64.01%	1.36%
Spelling performance (max score: 88)	88	57.22	1.48

### Stimuli

#### Learning materials.

**Fig 1:**
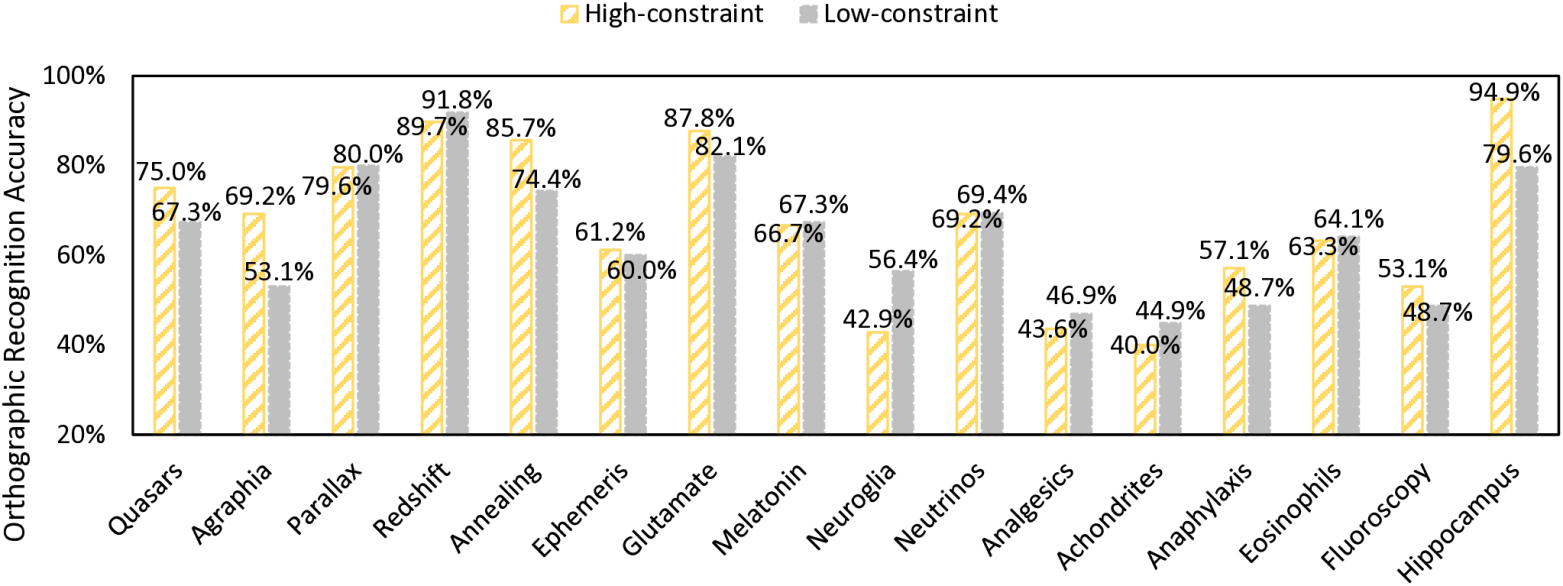
Orthographic recognition accuracy rates for each target word in order of word length.

Sixteen discipline-specific technical words were selected from four academic disciplines: astronomy, neurology, pharmacology, and physics (4 words per category, see Fig 1 for all target words). The target words were low-frequency English nouns of varying lengths (7–11 letters) that, on average, occurred 0.52 times per million in the Corpus of Contemporary American English [35]. Ten high-constraint sentences and ten low-constraint sentences were created for each target word. The word choice and construction in these sentences were typical of academic writing that may appear in a textbook. The high-constraint sentences related to the word’s core meaning (e.g., ***glutamate*** *must be paired with specific receptors on the receiving cell*), while the low-constraint sentences were true but vague (e.g., ***glutamate*** *can be artificial or naturally occurring*). Around 75% of the target words and sentences were adapted from Kuipers et al. [22], and minor changes were made to match stimuli properties across conditions.

The high- and low-constraint sentences were matched on readability, number of words, and lexical density (see Table 2 for the properties of the target words and sentences across conditions). For the words in each sentence, the cumulative lexical frequency based on subtitles (logged per million frequency in SUBTLEX-US; [36]) was also matched across conditions. These served to minimize the effects of the sentence stimuli on the learning difficulty of the target words. The lexical density of the sentences, operationalized by the proportion of content words over the total number of words was between 50.59% and 63.41%, which was on par with English news articles [37]. The Flesch reading ease of the sentences, a readability index weighted by the number of words and number of syllables of the words, varied from 32.12 to 39.77 and were considered college-level [38].

**Table 2:** Stimuli properties.

	Mean	Standard Error	Range
Target word length (no. of letters)	9.38	0.33	7–11
Target word frequency per million (Corpus of Contemporary American English)	0.52	0.17	0.002–2.602
Length of sentences (no. of words)			
High-constraint sentences	8.19	0.10	7.3–8.8
Low-constraint sentences	8.13	0.10	7.4–8.7
Lexical density			
High-constraint sentences	58.19%	1.00%	50.59% – 63.41%
Low-constraint sentences	56.67%	0.57%	53.49% – 62.07%
Flesch reading ease			
High-constraint sentences	35.07	0.46	32.12–38.98
Low-constraint sentences	35.52	0.55	32.41–39.77
Cumulative lexical frequency (logged per million frequency in SUBTLEX-US)			
High-constraint sentences	4.11	0.04	0.30–6.18
Low-constraint sentences	4.21	0.04	0.48–6.18

Each participant received one learning list of 160 sentences, which consisted of 80 high-constraint sentences of 8 target words and 80 low-constraint sentences of the other 8 target words (learning condition as a within-participants factor). So, the number of exposures for target words in the two conditions was the same. Four counterbalanced learning lists were created, each divided into four blocks. To approach more naturalistic learning contexts, each block included four target words from the same category: high-constraint sentences were presented for two target words while low-constraint sentences were presented for the other two. The order of the blocks and that of sentences within a block were randomized for each participant.

#### Testing materials.

The outcome measures focused on the receptive knowledge of target words’ orthography and semantics, operationalized as accuracy in multiple-choice questions. One orthographic recognition question was generated for each target word, resulting in 16 questions. The participants were asked to select the correct word spelling out of four options: (a) the correctly spelled target word (e.g., *ephemeris*), (b) a pseudoword with one letter deleted from or added to the target word (e.g., *ephemetris*), (c) a pseudoword with one letter of the target word replaced (e.g., *ephemeras*), and (d) a pseudoword combining both changes (e.g., *ephemetras*). The design of the answer choices discouraged guessing strategies because all choices had similar variations. However, the distance to the correct answer was different among the choices. The choices with one-letter differences were closer to the correct answer than those with two-letter differences and thus could reflect partial knowledge of the correct word form. All the pseudowords followed English spelling rules such that the test performance could reflect the quality of participants’ orthographic representations after word learning. To ensure that the learners’ choices were not confounded with spelling typicality in the English orthography, we compared the sum, mean, and positional bigram frequencies of the response options based on the English Lexicon Project database (https://elexicon.wustl.edu/). Results of the one-way ANOVAs confirmed that the learned items and distractor items (three levels: original, 1-letter difference, and 2-letter difference) did not differ significantly in their bigram frequencies (*p*s > .987), so correct responses should be based on the orthographic representations of the learned items rather than their orthographic similarity to existing English words. Data and detailed results of the supplementary analysis can be found on the OSF repository.

For semantic recognition, three questions were generated for each target word, giving a total of 48 semantic recognition questions. The original phrasings of the high-constraint sentences were modified to prevent the participants from relying on surface clues when making a response (e.g., *“Which should attach to specific receptors to work?”* for the high-constraint sentence *“Glutamate must be paired with specific receptors on the receiving cell”*). For each question, four answer options were selected from a counterbalanced target word list: (a) the correct answer (e.g., *glutamate*), (b) a distractor from the same category as the correct answer (e.g., *hippocampus*), and (c) two distractors from a different category (e.g., *redshift* and *neutrinos*). Again, the answer choices allowed for analyses of partial learning when the options were split into relatively subtle errors (options from the same category as target words) and obvious errors (options from a different category).

To objectively assess participants’ prior L2 abilities, we also administered the LexTALE [39] and a spelling recognition task [40]. The LexTALE is an unspeeded visual lexical decision task with 60 trials, which provides a reliable index of vocabulary breadth for ESL learners with medium to high proficiency. The task contains 40 English words that are 4–12 letters long with an average occurrence of 6.4 per million in the CELEX database [41] and 20 pseudowords that are orthographically legal and pronounceable (e.g., *proom*). The spelling recognition task measures spelling ability for common English words. Participants needed to identify 44 misspellings with mostly preserved pronunciations (e.g., *benafit*, *importent*) out of 88 items. The likelihood that college-level ESL participants had not acquired these common English words was minimal, so this spelling recognition task tapped the participants’ precise orthographic representation rather than their vocabulary knowledge of the tested items.

### Procedure

The instructions on the procedure were sent to the participants via email, and they were asked to complete the online tasks in a quiet and distraction-free environment. All data were collected through online platforms, including Qualtrics (https://www.qualtrics.com/) for language background, the LexTALE website (https://lextale.com/takethetest.html), and the E-Prime Go system (https://pstnet.com/eprime-go/) for the other learning and testing tasks. The data were then linked through anonymized participant codes.

In the learning phase, the participants learned the 16 English technical words through a self-paced reading paradigm [22]. Each trial started with a one-second presentation of the target word (e.g., *glutamate*) to ensure the same amount of target word exposure. Then, the rest of the high-constraint or low-constraint sentence was displayed, and the participants pressed a button when they finished reading. They then responded on a 1-to-5 scale of how well they knew the target word (i.e., confidence self-ratings, 5 being the highest) and continued to the next trial until they finished reading all the sentences. Short breaks were allowed between blocks, and one practice trial was used to familiarize the participants with the procedure initially. No distractor task was included between the learning and testing phases, because we aimed to test immediate word learning performance.

In the testing phase, the 16 orthographic recognition questions were presented first in a randomized order, and the participants indicated their answers by pressing “A,” “B,” “C,” or “D” on the keyboard as quickly and accurately as possible. The question remained on the screen until a response, and no feedback was given. Subsequently, the 48 semantic recognition questions were presented in random order and completed similarly. The learning and testing tasks typically took no more than 60 minutes in total, so participants’ tiredness was minimized.

### Data analysis

Among the participants who completed the online tasks as required, 9 were excluded due to extremely fast responses (mean reaction time shorter than one second) in one or both of the recognition tests, and the remaining 88 participants were included in the following analysis. After 5.4% of the trials were further discarded due to a programming mistake in one of the learning lists, descriptive analyses were used to examine participants’ confidence self-ratings during word learning and overall performance in the recognition tests. The participants’ responses to the orthographic and semantic recognition questions were categorized into correct and incorrect responses. Correct responses were considered to reflect full receptive knowledge, while incorrect responses were further divided into relatively subtle errors, reflecting partial knowledge, and obvious errors.

Trial-level accuracy data in orthographic recognition and semantic recognition (i.e., OrthACC, SemACC) were submitted separately to generalized linear mixed-effects modeling (GLMM) in R Version 3.5.3 [42], with the *glmer* function (binomial distribution). The *lmerTest* package [43] was used to calculate *p* values with Satterthwaite approximation. The fixed effects of the models included relative word length (rLength, i.e., the difference between target word length and the minimal word length, 7) and learning condition (Cond, sentences with high vs. low semantic constraint). Deviation coding (i.e., sum contrasts) was adopted for the sentence constraint condition. Each participant’s scores in the LexTALE vocabulary test and the spelling recognition test were transformed to *z* scores (zVocab and zSpell, respectively) and entered into the models to test how individual differences in prior L2 abilities affect the learning outcomes. Additionally, the interactions of relative word length, contextual support, and prior L2 abilities were included in the initial models.

For analyses of partial learning outcomes, we categorized the incorrect responses into two types: relatively subtle errors (coded as “1”) and obvious errors (coded as “0”). For orthographic recognition, relatively subtle errors were pseudowords with a one-letter difference (one letter being deleted, added, or replaced) from the target word, while obvious errors were pseudowords with two combined differences from the target word. For semantic recognition, relatively subtle errors were distractors from the same category as the target word, while obvious errors were distractors from a different category. In this second set of analyses, the error data in orthographic recognition and semantic recognition (i.e., OrthErr, SemErr) were submitted to GLMM analysis separately, with the same fixed effects entered as the above GLMM models.

In all the models, the random-effects structure included by-participant and by-item random intercepts as well as by-participant random slopes for Cond and rLength and by-item random slope for Cond. Therefore, the formulae for the initial models on orthographic recognition accuracy, orthographic recognition errors, semantic recognition accuracy, and semantic recognition errors were [OrthACC/OrthErr/SemACC/SemErr ~ Cond*rLength*zVocab + Cond*rLength*zSpell + (1 + Cond + rLength | participant) + (1 + Cond | word)]. For each model, likelihood ratio tests were conducted to remove interaction effects that did not significantly improve model fit [44]. The model simplification steps were documented in the R script available from: https://osf.io/fxqvr/?view_only=f4ae94c1230148c684154063f30dd913.

## Results

### Confidence self-ratings and overall performance

The participants read ten high- or low-constraint sentences to learn each of the 16 English technical words and indicated how well they knew the target word on a 1-to-5 scale after reading each sentence. The mean confidence self-ratings in the high- and low-constraint conditions were 3.70 (*SE* = 0.06) and 3.17 (*SE* = 0.09), respectively, across the ten sentences. In the high-constraint condition, the mean rating score increased from 3.13 (*SE* = 0.08) after the first sentence to 3.95 (*SE* = 0.08) after the tenth sentence. In the low-constraint condition, the mean rating score increased from 2.74 (*SE* = 0.09) after the first sentence to 3.38 (*SE* = 0.10) after the tenth sentence. The confidence self-ratings were significantly higher in the high-constraint condition than the low-constraint condition for all ten sentences (*p*s < .001), suggesting successful manipulation on the level of contextual support. On the other hand, the mean rating scores after reading the first sentence for each target word were relatively low in both conditions (i.e., 3.13 and 2.74). The rating scores gradually increased with more exposures but did not get close to the highest confidence level (i.e., 5) after the tenth sentence. These results corroborated the incremental nature of the word learning process [12,33,34] as well as the relatively low quality of L2 lexical representations during initial acquisition [32], which validated the use of the self-paced reading paradigm [22] in studying initial acquisition of L2 technical words.

**Fig 2:**
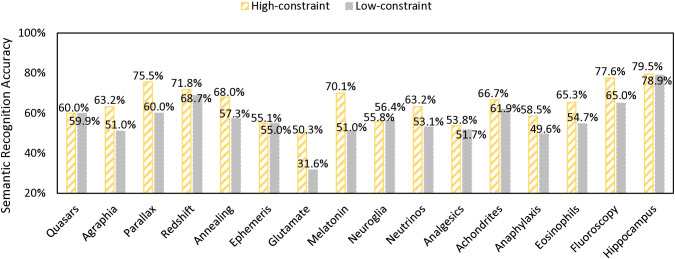
Semantic recognition accuracy rates for each target word in order of word length.

In the testing phase, the participants made 881 correct responses among a total of 1,332 valid trials in the orthographic recognition task (66.1%, see Fig 1 for individual target words’ recognition accuracy). In the error analysis for orthographic recognition, the participants made relatively subtle errors (i.e., selecting the distractors with one letter different from the target word) in 354 trials out of 451 incorrect responses (78.5%). In the semantic recognition task, the participants made 2,427 correct responses among a total of 3,996 valid trials (60.7%, see Fig 2). In the error analysis for semantic recognition, the participants made relatively subtle errors (i.e., selecting the distractors from the same category as the target word) in 643 trials out of 1,569 incorrect responses (41.0%).

### Orthographic recognition

The model simplification procedure retained the interaction between word length and L2 vocabulary knowledge (*p* = .002). The left panel of Table 3 shows the parameter estimates and *p* values of the fixed effects on orthographic recognition accuracy. Contextual support (i.e., word learning with semantically high-constraint vs. low-constraint sentences) had no significant effect on orthographic recognition (*p* = .249). Word length showed a significant detrimental effect on orthographic recognition (*p* = .047), suggesting that longer word forms were more difficult to recognize than shorter ones. General L2 spelling skill was positively related to orthographic recognition performance (*p* = .015). Moreover, L2 vocabulary was also associated with better orthographic recognition (*p* = .001), but the relation between these two variables became weaker as the word length increased (*p* = .002). In other words, individuals with a larger L2 vocabulary learned the orthographic aspect of L2 technical words better and this advantage was greater for shorter than longer words.

**Table 3:** Parameter Estimates, Standard Errors, Confidence Intervals, and Statistical Significance in the GLMM Analyses of Orthographic Recognition Performance.

Fixed Effects	Orthographic Recognition Accuracy (OrthACC: 1 – correct; 0 – incorrect)					Orthographic Recognition Error Type (OrthErr: 1 – relatively subtle; 0 – obvious)				
	*β*	*SE*	Wald 95% CI	*z*	*p*	*β*	*SE*	Wald 95% CI	*z*	*p*
Intercept	1.76	0.47	0.84, 2.69	3.73	<.001***	1.92	0.34	1.26, 2.59	5.65	<.001***
Cond1	0.09	0.08	−0.06, 0.23	1.15	.249	0.16	0.12	−0.08, 0.4	1.33	.182
rLength	−0.32	0.16	−0.65, 0	−1.98	.047*	−0.20	0.11	−0.42, 0.02	−1.76	.079
zVocab	0.81	0.25	0.33, 1.3	3.28	.001**	−0.13	0.13	−0.37, 0.12	−1.01	.314
zSpell	0.33	0.14	0.06, 0.61	2.42	.015*	0.29	0.13	0.04, 0.54	2.24	.025*
rLength*zVocab	−0.22	0.07	−0.35, −0.08	−3.07	.002**	--	--	--	--	--
**Random Effects**		**Variance**	***SD***	**Correlation**			**Variance**	***SD***	**Correlation**	
Participants	Intercept	2.35	1.53				0.02	0.14		
	Cond	0.02	0.13	−0.52			0.01	0.09	1.00	
	rLength	0.12	0.34	−0.76	−0.16		0.001	0.04	−1.00	−1.00
Words	Intercept	0.68	0.82				0.06	0.25		
	Cond	0.01	0.11	1.00			0.003	0.05	−1.00	
	OrthACC ~ Cond + rLength*zVocab + zSpell + (1 + Cond + rLength | participant) + (1 + Cond | word)					OrthErr ~ Cond + rLength + zVocab + zSpell + (1 + Cond + rLength | participant) + (1 + Cond | word)				

*Note*. Deviation coding was adopted for Cond (learning condition: low-constraint and high-constraint sentences). rLength (relative word length) = number of letters in the target word – 7 (the minimal word length). zVocab and zSpell were *z* scores of each participant’s general L2 vocabulary knowledge and spelling skill.

* *p* < .05, ** *p* < .01, *** *p* < .001.

**Fig 3:**
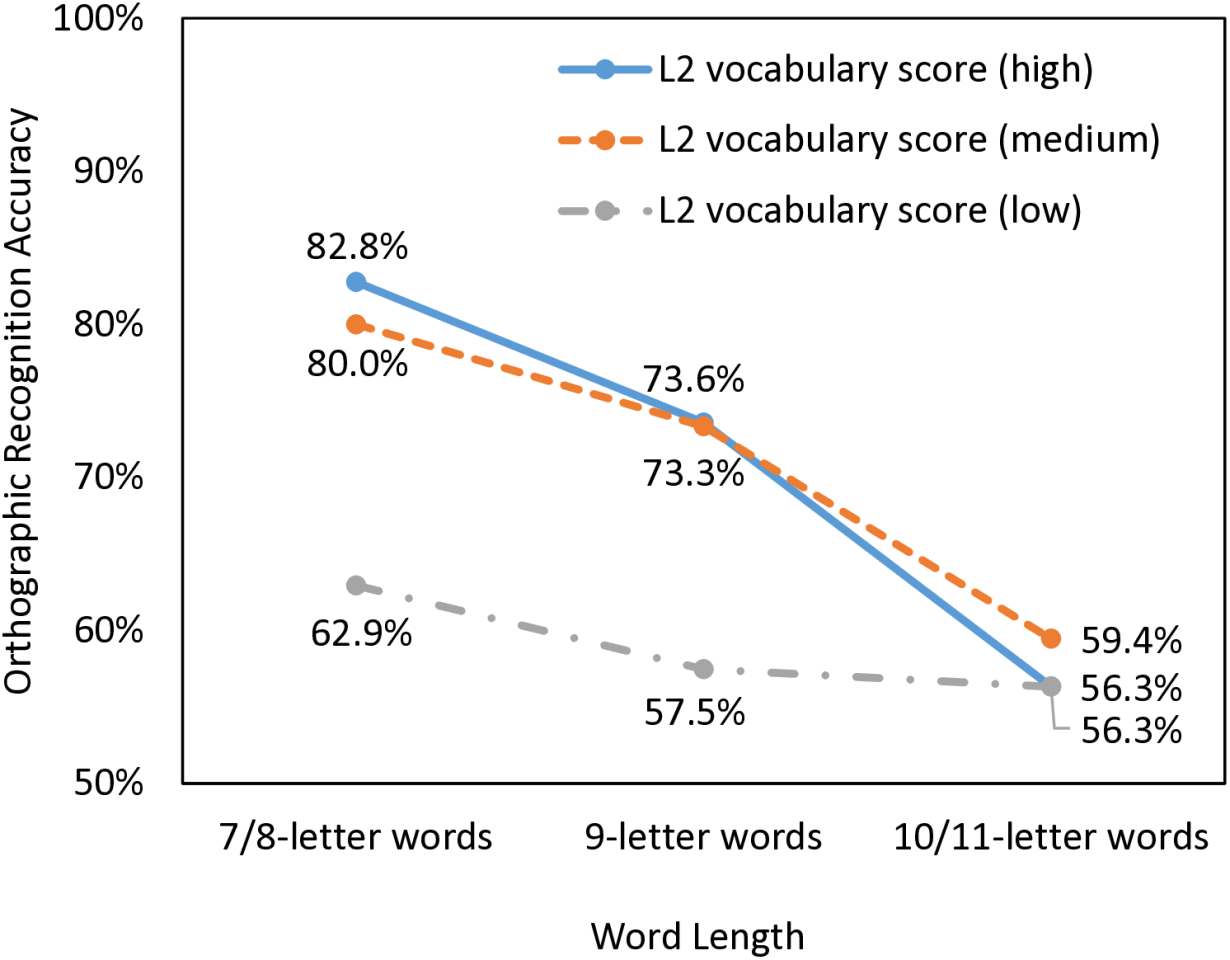
Orthographic recognition accuracy as a function of word length and L2 vocabulary knowledge.

To further visualize the interaction effect between word length and L2 vocabulary knowledge, we divided the participants into three levels based on their L2 vocabulary scores (high: zVocab > 0.3, *N* = 29; medium: 0.3 ≥ zVocab ≥ −0.5, *N* = 30; low: zVocab < −0.5, *N* = 29); the target words were also divided into 7/8-letter, 9-letter, and 10/11-letter words (*N*s = 4, 6, 6, respectively). Fig 3 displays the orthographic recognition accuracy as a function of word length and L2 vocabulary. There appeared to be a positive impact of L2 vocabulary knowledge for the shorter words, whereas prior L2 vocabulary knowledge did not impact orthographic learning of longer words. Pairwise comparisons showed that the high- and medium-vocabulary groups performed similarly across different levels of word length (*p*s > .601). Both groups performed significantly better than the low-vocabulary group for 7/8-letter and 9-letter words (*p*s < .022), but not for 10/11-letter words (*p*s > .692). At the same time, the accuracy difference between 7/8-letter and 9-letter words was significant in the high-vocabulary group (*p* = .026) only, while that between 9-letter and 10/11-letter words was significant in the high- and medium-vocabulary groups but not the low-vocabulary group (*p*s = .001,.011,.874, respectively).

The percentage of relatively subtle errors over all incorrect responses was taken as a supplementary learning outcome, with a higher percentage indicating better performance. All interaction effects in the model of orthographic recognition errors were non-significant and removed. As shown in the right panel of Table 3, word length had a non-significant detrimental effect on the percentage of relatively subtle errors over all incorrect responses (*p* = .079). L2 spelling skill predicted more partial recognition of word orthography (*p* = .025).

### Semantic Recognition

The model of semantic recognition accuracy did not retain any interaction effects after model simplification. The left panel of Table 4 shows the parameter estimates and *p* values of the fixed effects on semantic recognition accuracy. L2 technical words learned through semantically high-constraint sentences elicited better performance than the low-constraint condition (*p* < .001), while target word length, general L2 vocabulary knowledge, and L2 spelling skill did not significantly contribute to semantic recognition accuracy.

**Table 4:** Parameter Estimates, Standard Errors, Confidence Intervals, and Statistical Significance in the GLMM Analyses of Semantic Recognition Performance.

	Semantic Recognition Accuracy (SemACC: 1 – correct; 0 – incorrect)					Semantic Recognition Error Type (SemErr: 1 – relatively subtle; 0 – obvious)				
Fixed Effects	*β*	*SE*	Wald 95% CI	*z*	*p*	*β*	*SE*	Wald 95% CI	*z*	*p*
Intercept	0.29	0.26	−0.21, 0.79	1.12	.261	−0.84	0.22	−1.27, −0.41	−3.85	<.001***
Cond1	0.20	0.05	0.11, 0.29	4.29	<.001***	−0.06	0.06	−0.18, 0.06	−0.96	.340
rLength	0.10	0.09	−0.08, 0.28	1.06	.291	0.19	0.08	0.04, 0.35	2.42	.016*
zVocab	0.11	0.10	−0.09, 0.32	1.12	.264	0.05	0.07	−0.08, 0.19	0.82	.410
zSpell	0.09	0.10	−0.11, 0.29	0.89	.373	0.00	0.06	−0.12, 0.12	0.00	.997
**Random Effects**		**Variance**	***SD***	**Correlation**			**Variance**	***SD***	**Correlation**	
Participants	Intercept	0.35	0.59				0.003	0.06		
	Cond	0.01	0.11	−0.53			0.005	0.07	1.00	
	rLength	0.02	0.15	0.99	−0.40		0.003	0.06	1.00	1.00
Words	Intercept	0.20	0.44				0.15	0.38		
	Cond	0.01	0.10	−0.16			0.01	0.11	0.97	
	SemACC ~ Cond + rLength + zVocab + zSpell + (1 + Cond + rLength | participant) + (1 + Cond | word)					SemErr ~ Cond + rLength + zVocab + zSpell + (1 + Cond + rLength | participant) + (1 + Cond | word)				

*Note*. Deviation coding was adopted for Cond (learning condition: low-constraint and high-constraint sentences). rLength (relative word length) = number of letters in the target word – 7 (the minimal word length). zVocab and zSpell were *z* scores of each participant’s general L2 vocabulary knowledge and spelling skill.

*p < .05, **p < .01, ***p < .001.

The model of semantic recognition errors did not include any interaction effects either. As shown in Table 4, word length had a significantly positive relation with the percentage of relatively subtle errors over all incorrect responses (*p* = .016). All other effects, including the main effect of contextual support, were non-significant.

## Discussion

The current study examined how word length, contextual support, and prior L2 skills affect Chinese-English university students’ orthographic and semantic learning of English technical words. These L2 English learners read sentences with high or low semantic constraints to learn English technical words of varying length and then completed recognition tests. The overall accuracy rate was 66.1% for orthographic recognition and 60.7% for semantic recognition, showing that the learners successfully acquired the L2 technical words through the self-paced reading paradigm [22]. However, the modest learning gains reflected relatively low quality of lexical representations during initial acquisition of L2 technical words. Contrastive results on full and partial learning outcomes showed that word length, contextual support, and prior L2 skills had different impacts on learning the orthography and semantics of L2 technical words.

### Word length effects *on* L2 technical word learning

The current study found a negative main effect of word length on overall orthographic recognition accuracy, which aligned with previous findings that longer word forms are more difficult to learn and remember [12–13]. When the participants were divided into three levels based on their L2 vocabulary scores, significant word length effects were found in high- and medium-vocabulary groups only but not the low-vocabulary group. The current orthographic recognition task contained subtle differences (i.e., 1–2 letters) and required high lexical quality to distinguish between answer choices [30]. The high- and medium-vocabulary groups performed well for 7/8-letter words, but they might potentially experience cognitive overload when learning longer words, resulting in lower-quality orthographic representations. In contrast, the low-vocabulary group performed poorly for all target words of varying length, suggesting the presence of a floor effect.

Longer words did not show more partial orthographic learning than shorter words, contradicting our prediction and the results of Barcroft and Rott [12]. Over half of our target words (9 out of 16) had 4 or more syllables, while Barcroft and Rott focused on 2- and 3-syllable words, so our stimuli generally contained longer and more complex words. For longer words, it was more difficult to accurately retrieve all letters when deciding between similarly spelled choices, because it incurred a higher cognitive load to match all letters [17–18]. Longer words also left more room for spelling errors to be undetected in recognition because 2-letter spelling errors were proportionally smaller in 11-letter than 7-letter words.

A positive relation between word length and partial semantic recognition was observed, but not for full semantic recognition, suggesting that word length did not directly affect semantic encoding. Our L2 technical words tended to be conceptually difficult and the orthographic parts (e.g., morphemes) did not contain transparent semantic information that might facilitate semantic recognition. However, longer words might have more distinctive phonological or orthographic features that allowed participants to more easily associate them with a broad category (i.e., partial semantic recognition). Category information relies on semantic clustering, which may benefit from salient cues that longer words tend to provide. Meanwhile, definitions demand fine-grained semantic encoding, and surface-level cues such as word shape or number of letters may be insufficient to facilitate recognition of fine-grained semantic information in the definition sentences. This would be partly consistent with the previous finding that longer word length facilitated semantic recall after exposure to teacher speech [19]. Importantly, the current study showed the word length effect on learning L2 semantics through academic reading, beyond the auditory or audiovisual modalities.

### Effects *of* Contextual Support *on* L2 Technical Word Learning

Contextual support showed no effect on full and partial orthographic recognition. This agreed with Yi et al.’s report of a null effect of contextual support on orthographic recognition [26]. Although this study employed a longer learning paradigm than Yi et al., with ten sentences for each target word, the semantic constraint of the sentence context did not enhance learners’ orthographic recognition performance. Previous studies by Barcroft [28–29] used the presence or absence of a semantic elaboration task to show that if cognitive resources were allocated to semantic processing, there would be detrimental effects on orthographic learning of general L2 words. Further investigation is needed to examine whether such effects would replicate in L2 technical word learning, as our study did not employ a task requiring explicit semantic elaboration.

Meanwhile, we expected that L2 technical words learned via high-constraint sentences would result in better semantic recognition. Results indeed indicated a strong effect of contextual support on semantic recognition accuracy, consistent with previous studies [15,23,24]. Although the learners were initially unfamiliar with the meaning of these technical words, reading constraining sentences was sufficient to allow them to answer semantic recognition questions [22]. In contrast, contextual support had no significant effect on partial semantic learning (i.e., selecting another target word from the same discipline). We employed a blocked presentation sequence that showed all target words from the same discipline within one block regardless of contextual condition. Thus, partial semantic learning as measured in the current study might reflect the extent to which the newly learned words from the same discipline were associated with each other in a semantic network instead of the quality of the individual word’s semantic representation. The factor of contextual support did not interact with word length or either measure of L2 proficiency to influence learning performance. This was in line with the notion that semantic learning of L2 technical words mainly benefits from explicit definitions and contextualized learning [8,10,14].

### Effects *of* L2 Proficiency *on* L2 Technical Word Learning

We hypothesized that L2 spelling skill would predict orthographic recognition accuracy after learning L2 technical words since the spelling task and the orthographic recognition task appeared to tap the same skills. As predicted, L2 spelling skill was positively related to full and partial orthographic recognition performance in L2 technical word learning. For ESL learners, sensitivity to L2 word forms may lead to successful orthographic segmentation of phonological units (i.e., phonemes and syllables) and morphological units (i.e., affixes). This kind of chunking would serve to reduce the cognitive load and may facilitate the establishment of orthographic representations and the recognition of newly learned word forms. General L2 spelling skill was associated with a greater ability to reject obvious spelling errors. This may relate to the learners’ pattern recognition abilities, which lets them make educated guesses even if their answers are wrong. Skilled spellers may also rely on visual memory and other metacognitive strategies to aid the learning of new spellings, as spelling skill is a good index of general language abilities [40].

Interestingly, participants with larger L2 vocabulary size were also more successful in recognizing the spellings of novel L2 words. Learners with larger L2 vocabularies may be more familiar with orthographic patterns or knowledge of word roots, allowing them to form stable orthographic representations for the novel L2 words. However, the cognitive load in processing longer words appeared to constrain the facilitation, as supported by the interaction between L2 vocabulary breadth and word length. The distribution of subtle errors among incorrect responses also aligned with a cognitive load account since learners with larger L2 vocabularies did not have better partial orthographic recognition. Another possible explanation for the relation between L2 vocabulary and orthographic recognition accuracy is that the participants with larger L2 vocabularies were more likely to have known the target words before participating in this study. This explanation, however, is not plausible as L2 vocabulary did not predict semantic recognition accuracy in a similar manner. Therefore, these participants’ better orthographic recognition indeed reflected their larger learning gains rather than prior word knowledge.

Neither L2 vocabulary nor spelling skill significantly affected semantic recognition after learning L2 technical words, which agreed with existing reports [25,31]. This finding highlighted the importance of conceptual processing in learning L2 technical words, beyond the role of language proficiency. The technical words in the current study were selected from four academic disciplines of science: astronomy, neurology, pharmacology, and physics. The meanings of these words were relatively complex, and the learners needed to create coherent new concepts by connecting different pieces of information from multiple sentences. Thus, information processing at the conceptual level, in addition to language processing, might be essential during conceptually-mediated word learning [22], which could differentiate L2 technical word learning from general L2 word acquisition.

### Pedagogical implications

This study has practical implications for the teaching and learning of L2 technical words. First, word length negatively influenced orthographic recognition. Thus, instructors may provide additional support to help students tackle the difficulties of spelling long words. For example, they may leverage the visual or auditory distinctiveness of longer words to develop mnemonics. Second, illustrations of the technical words’ meanings from context would facilitate their learning and memory. Subject instructors could give definitions and ample contextualized information to strengthen the semantic component of word learning. If learners are exposed to novel L2 technical words outside the classroom, they could seek a clear definition by looking up the dictionary or checking a glossary to improve the initial learning of these words. Third, instructors may design answer options of multiple-choice questions to reflect partial knowledge when assessing vocabulary learning. Our participants showed evidence of partial learning, as their choices for relatively subtle errors were higher than chance. A gradation of difficulty in the distractors can help map the learning progress even if students choose wrong answers on a recognition task. Finally, learners’ general L2 vocabulary and spelling skills significantly predicted orthographic learning of L2 technical words, suggesting that strengthening general L2 proficiency could potentially enhance orthographic learning performance.

### Limitations and future directions

The first limitation of the current study was the relatively heterogeneous academic backgrounds of the sample, as we recruited university students with different majors. While we ensured that the majors did not coincide with the chosen disciplines, there would be natural variation in how familiar or interested participants felt toward certain disciplines. Future studies can screen participants on their disciplinary knowledge and interests by collecting self-report ratings, as these factors may affect learning effectiveness. Second, we did not have measures of participants’ cognitive skills, such as sustained attention and short-term memory, which may moderate the learning process, nor did we include attention checks within the learning protocol. However, these potential confounding factors were mitigated by modeling the by-participant random effects in the statistical analyses. Third, the current sample size was constrained by the resources available when the study was conducted, instead of being based on an *a priori* power analysis. Since a post-hoc power analysis may not be helpful in interpreting the results, we chose not to conduct the analysis. Fourth, the target words of the current study were technical words in different disciplines of pure science. Future studies may extend the study of word length and contextual support effects to the learning of technical words from other disciplines and general academic vocabulary which may be used across different disciplines. Fifth, we used behavioral learning outcomes as indicators of processing difficulties. It would be ideal to triangulate the findings in future research using self-reports of mental effort or physiological measures such as pupillometry or event-related brain potentials.

Finally, we noted that word length was not the only orthographic factor that might influence word learning. Visual inspection of Fig 1 showed that the idiosyncratic morphological or orthographic structure of the target words might have contributed to the orthographic recognition accuracy. The high accuracy rates for “redshift” and “hippocampus” could be due to the easily segmented word parts. Even though participants saw all target words the same number of times, these words contained easily recognizable morphemes that might have facilitated orthographic recognition. Given the relatively small number of target words in the current learning paradigm, we chose to focus on the salient orthographic factor of word length among the 16 learned items to retain sufficient statistical power. Future studies may manipulate various characteristics of the word stimuli and obtain ratings of perceived learning difficulty to tease apart the effects of word length and other word characteristics.

## Conclusions

The current study systematically investigated the effects of word length, contextual support, and L2 proficiency on different aspects of L2 technical word learning, with the same participants and learning protocol. Partial learning in recognition performance served as a supplementary learning outcome, which added to a fuller picture of L2 word learning. Results showed that longer words inhibited orthographic recognition but facilitated partial semantic recognition. Strong contextual support during initial acquisition of L2 technical words enhanced semantic recognition and did not affect orthographic recognition. Prior L2 skills (i.e., vocabulary breadth and spelling skill) were associated with better orthographic learning but not semantic learning of L2 technical words. The study revealed both item-related and learner-related effects influencing the orthographic and semantic recognition of recently learned L2 technical words, with implications for learners to adopt directed strategies to support L2 technical word learning.
